# The L10P Polymorphism and Serum Levels of Transforming Growth Factor β1 in Human Breast Cancer

**DOI:** 10.3390/ijms140815376

**Published:** 2013-07-24

**Authors:** Eva Taubenschuß, Erika Marton, Maurice Mogg, Barbara Frech, Lisa Ehart, Dana Muin, Martin Schreiber

**Affiliations:** 1 Department of Obstetrics and Gynecology, Medical University of Vienna, Waehringer Guertel 18-20, A-1090 Vienna, Austria; E-Mails: eva.taubenschuss@gmx.at (E.T.); erika.marton@meduniwien.ac.at (E.M.); maurice.mogg@gmail.com (M.M.); barbara.frech@gmx.net (B.F.); lisa.ehart@gmx.at (L.E.); dana.muin@meduniwien.ac.at (D.M.); 2 Comprehensive Cancer Center (CCC), Medical University of Vienna, A-1090 Vienna, Austria

**Keywords:** breast cancer, TGFβ1, rs1800470, L10P SNP, serum levels

## Abstract

The L10P single nucleotide polymorphism (SNP) is located in the signal sequence of the transforming growth factor β1 (*TGFβ*1) gene. The proline-encoding (Pro-) allele of this SNP has been associated with an increased breast cancer risk, which has been attributed to the elevated secretion of this TGFβ1 variant observed *in vitro* and in male subjects. Here we investigated the association of the L10P SNP with serum levels of TGFβ1 in female breast cancer patients and controls. We genotyped the L10P SNP in 276 breast cancer patients and 255 controls. Serum TGFβ1 concentrations were measured by enzyme-linked immunosorbent assay (ELISA) in a subset of the study population (*n* = 211). We found no evidence for an association of the L10P SNP with breast cancer risk (per-allele odds ratio: 0.91; 95% confidence interval: 0.71–1.16). However, patients with the Pro/Pro genotype exhibited a significantly younger age at breast cancer onset (55.2 ± 14.3 years) than Leu/Leu patients (60.6 ± 13.6 years; *p* = 0.04), which may reflect the ability of TGFβ to promote tumor progression. Mean TGFβ1 serum levels of Pro-allele carriers were 39.4 ± 7.4 ng/mL, whereas those of Leu/Leu subjects were 37.6 ± 6.0 ng/mL (*p* = 0.07). Thus, compared to a previous study of male subjects, we observed only a modest increase, if any, in TGFβ1 levels of female Pro-allele carriers.

## 1. Introduction

Transforming growth factor β (TGFβ) is a multifunctional cytokine and a key growth suppressor in many cell types, eliciting potent anti-proliferative and apoptotic responses [1–3]. Accordingly, loss-of-function mutations in the TGFβ pathway have been frequently observed in human tumors, classifying it as a tumor suppressor. However, at later stages of cancer progression tumor cells often develop resistance to the tumor suppressive activity of TGFβ. In tumor cells that are thus relieved from its growth inhibitory function, TGFβ can actively promote tumor progression, particularly by enhancing the invasiveness and metastatic propensity [2,4,5]. Whereas this dual role of TGFβ as a tumor suppressor and pro-tumorigenic factor is well understood in various model systems, it is less clear if and at which stage this switch from anti- to pro-tumorigenic activity of TGFβ occurs in human breast cancer.

The gene encoding TGFβ1, one of the three human TGFβ isoforms, contains a single-nucleotide polymorphism (SNP) at position 29 of its coding sequence, the major allele encoding leucine and the minor allele encoding proline as amino acid 10 (Leu10Pro; T + 29C; rs1800470; hereafter referred to as L10P). A large study of the Breast Cancer Association Consortium (BCAC) has reported an association of the Pro-allele with a moderate, but significantly increased, breast cancer risk (e.g., Pro/Pro *vs.* Leu/Leu: OR, 1.16; 95% confidence interval, 1.08–1.25 [6]). Other studies have either reported an increased risk, an unaltered risk, or even a decreased risk associated with the Pro-allele [7–24]. Shin *et al.* [19] suggested that these inconsistent results are due to the dual role of TGFβ, and that the Pro-allele may reduce the incidence of early-stage breast cancer, but promote the progression of late-stage breast cancer. In their study the Pro-allele was associated with a decreased risk of early-stage (0 or I), but a (non-significantly) increased risk of advanced-stage (III or IV) breast cancer [19]. The association of other *TGFβ*1 SNPs with breast cancer has also been analysed, such as the promoter SNPs C-509T and G-800A and the R25P coding SNP [7–13].

The L10P SNP is located in the signal sequence of *TGFβ*1, and has been suggested to affect the efficiency of TGFβ1 secretion [7]. Transfections of HeLa cells with expression vectors encoding the Pro-allele resulted in a more than two-fold higher secretion of TGFβ1 into the culture medium than parallel transfections with the Leu-allele [7]. Accordingly, the Pro-allele is considered a “high-activity” (hypermorphic) allele compared to the Leu-allele. The *in vivo* association of the L10P genotype with TGFβ1 serum levels has been investigated in a Japanese study of human myocardial infarction [25]. In this study, the serum concentrations of TGFβ1 were significantly higher in male patients and controls with the Pro/Pro *vs.* the Leu/Leu genotype. However, correlations of TGFβ1 serum levels with L10P genotypes in female patients or controls have not been not reported so far [25].

## 2. Results and Discussion

### 2.1. The *TGFβ1* L10P SNP and Breast Cancer Risk

The L10P SNP of *TGFβ*1 was genotyped in 274 breast cancer patients and 252 female controls. The frequencies of the L10P genotypes in patients and controls, and clinical characteristics of the patients are shown in Table 1. The frequency of the Pro-allele was 40.0% in patients and 42.3% in controls.

The controls and the breast cancer patients were both in Hardy-Weinberg equilibrium (*p* = 0.50 and *p* = 0.96 respectively). The Pro/Pro genotype was slightly less frequent in breast cancer patients than in controls (16.1% and 19.0%, respectively; Table 1). On the other hand, the fraction of patients with the Pro/Pro genotype tended to be increased in several patient subgroups associated with advanced cancer progression and/or poor prognosis. For example, 19.3% of grade 3 *vs.* 14.6% of grade 1/2; 24.6% of estrogen receptor (ER) negative *vs.* 12.9% of ER positive; and 19.7% of progesterone receptor (PR) negative *vs.* 13.0% of PR positive patients exhibited the Pro/Pro genotype (Table 1). However, none of these differences in genotype distribution among clinical subgroups were statistically significant (Table 1).

**Figure 1:**
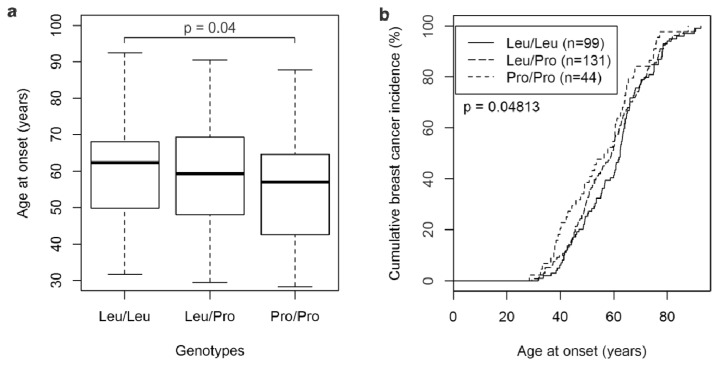
Breast cancer patients with the *TGFβ*1 Pro/Pro genotype exhibit a younger age at onset. (**a**) Boxplot of the age at diagnosis of patients with genotypes Leu/Leu (*n* = 99), Leu/Pro (*n* = 131), and Pro/Pro (*n* = 44); (**b**) Curves of the cumulative breast cancer incidence at the indicated ages at onset of patients with genotypes Leu/Leu, Leu/Pro, and Pro/Pro.

Next, odds ratios and 95% confidence intervals for breast cancer risk were determined. All comparisons revealed odds ratios close to unity, and any deviations from unity were not significant (Table 2). Thus, the Pro/Pro genotype, like the Pro-allele, was not associated with an increased breast cancer risk. We next analyzed the association of the L10P genotype with the age at breast cancer diagnosis. Interestingly, the mean age at breast cancer onset of patients with the Pro/Pro genotype was 55.2 ± 14.3 years (median age, 57.0 years), whereas that of Leu/Leu patients was 60.6 ± 13.6 years (median age, 62.4 years; *p* = 0.04; Figure 1A). Leu/Pro patients were diagnosed at an intermediate mean age (58.5 ± 14.1 years; median age, 59.3 years; Figure 1A). Comparison of the cumulative breast cancer incidence of all three genotypes by a log rank test also revealed significant differences (*p* = 0.048; Figure 1B).

### 2.2. Analysis of TGFβ1 Serum Levels

**Figure 2:**
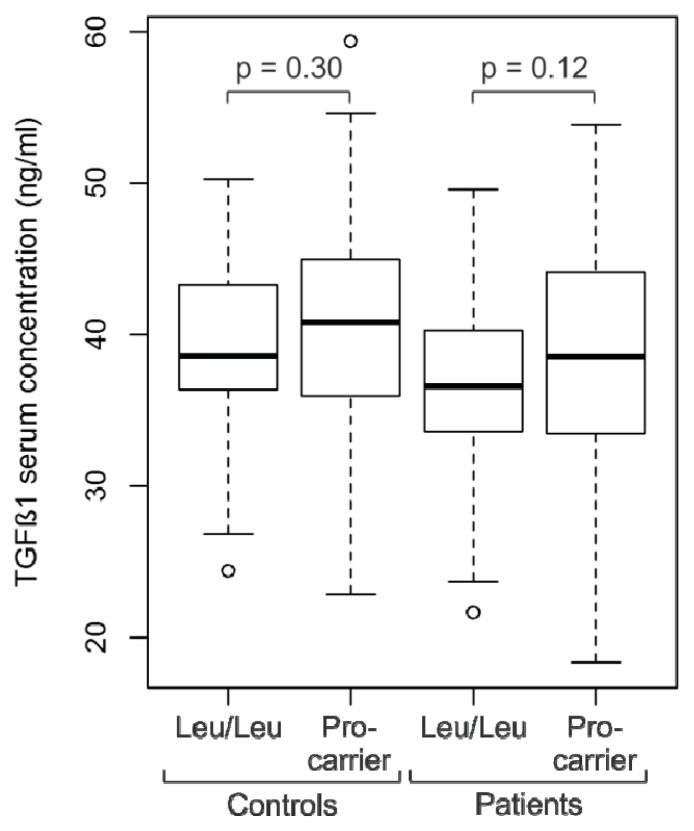
Boxplot of TGFß1 serum concentrations in patients and control subjects with the Leu/Leu genotype, and the Leu/Pro or Pro/Pro genotype (Pro-carrier).

Next, serum levels of TGFβ1 protein were analyzed in breast cancer patients (*n* = 110) and control subjects (*n* = 101) with known L10P genotypes (Figure 2). We measured TGFβ1 serum concentrations of 37.9 ± 7.1 ng/mL in the patients, which was significantly different from the levels measured in the controls (39.8 ± 6.8 ng/mL; *p* = 0.048). Patients with the Leu/Leu genotype (*n* = 36) had mean TGFβ1 serum levels of 36.5 ± 6.0 ng/mL, whereas mean serum levels of Pro-carriers (Leu/Pro or Pro/Pro; *n* = 74) were 38.6 ± 7.4 (*p* = 0.12; Figure 2). In the control population, mean TGFβ1 levels of Leu/Leu individuals (*n* = 33) were 38.8 ± 5.8 ng/mL, and of Pro-carriers (*n* = 68) 40.3 ± 7.3 ng/mL (*p* = 0.30). Thus, Pro carriers had slightly higher mean TGFβ1 serum concentrations than Leu/Leu individuals both in the control group and in the breast cancer patients, although the observed differences were not significant (Figure 2). When patients and control subjects were combined, mean TGFβ1 levels of Leu/Leu individuals (*n* = 69) were determined to be 37.6 ± 6.0 ng/mL, and those of Pro carriers (*n* = 142) were 39.4 ± 7.4 ng/mL (*p* = 0.07). Furthermore, no significant differences in TGFβ1 serum levels were observed between patients with ER negative *vs.* ER positive, ductal *vs.* lobular, pN0 *vs.* pN+, pT1 *vs.* pT2-4 tumors, or between patients younger or older than 55 years (data not shown).

### 2.3. Discussion

The L10P SNP of *TGFβ*1 has been extensively studied, and is characterized by contradictory reported results: some studies showed an increased breast cancer risk associated with the Pro-allele [6–9], others a decreased risk [10], and yet others—including this study (Table 2)—have found no evidence of a significantly altered risk [11–20]. These inconsistent results have been attributed to the unique dual role of TGFβ, which is thought to act as a tumor suppressor during early cancer stages, but to promote cancer progression at late stages [1,2,4,19]. Whereas there is extensive evidence for this dual role of TGFβ in model systems, it is less clear at which stage this switch from tumor suppressor to progression factor occurs in human breast cancer and in which tumor subclass(es) it has already occurred at the time of diagnosis [2,3,19]. It has been suggested that the Pro-allele is associated with a reduced risk of *in situ* tumors, but an increased risk of invasive breast cancer; or with a reduced risk of early-stage invasive breast cancer, but an increased risk of breast cancer with advanced stages [6,19].

The L10P Pro-allele has been suggested to be a hypermorphic (high-activity) allele of *TGFβ*1. A proline residue at position 10, which is located in the signal sequence of *TGFβ*1, is thought to increase the efficiency of TGFβ1 secretion [7]. Expression of the Pro-allele resulted in a more than two-fold higher secretion of TGFβ1 than expression of the Leu-allele in *in vitro* transfection experiments [7]. The *in vivo* serum concentrations of TGFβ1 were significantly higher in male myocardial infarction patients and controls with the Pro/Pro genotype than in subjects with the Leu/Leu genotype (>50 *vs.* <40 ng/mL), whereas TGFβ1 serum levels in female patients or controls were not reported [25]. Interestingly, in this study the Pro-allele was significantly more frequent in male, but not in female patients compared to controls, suggesting a differential effect of the L10P genotype in male *vs.* female subjects [25]. In a gastric cancer study, TGFβ1 serum levels did not correlate with L10P genotypes [26]. To the best of our knowledge, our study is the first to correlate L10P genotypes with TGFβ1 serum levels in breast cancer patients and exclusively female controls. We observed moderately elevated serum levels in patients and controls with one or two Pro-alleles, although this trend was not significant at the *p* < 0.05 level (Figure 2). Collectively, the findings by us and others indicate that the Pro-allele may also lead to higher TGFβ1 secretion *in vivo*, but that the observed effects on serum levels are less pronounced and more heterogeneous than *in vitro*.

We hypothesize that, if the Pro-allele is indeed hypermorphic it should be associated with a reduced overall breast cancer incidence, due to the tumor suppressor activity of TGFβ initially effective in all tumors during the early phases of the multi-step progression. This is reflected by odds ratios below unity reported by some studies, and a particularly pronounced reduction in breast cancer risk in a prospective study (Pro/Pro *vs.* Leu-carriers: HR, 0.36; 95% confidence interval (c.i.), 0.17–0.75 [10]). On the other hand, in tumors that do eventually develop, the Pro-allele is predicted to be associated with an advanced, invasive, and metastatic disease, due to the ability of TGFβ to promote tumor progression at later stages by modulating the tumor microenvironment, enhancing invasiveness, and inhibition of immune cell function [1–4]. Indeed, patients who carried the Pro-allele had a significantly reduced 5-year disease-free survival rate compared to Leu/Leu patients [9]. In association studies, this effect should be reflected by odds ratios above unity in subpopulations with advanced stages, and in entire studies of patients with a predominantly advanced cancer stage.

How is the “advanced stage”, in which tumors are responsive to the tumor promoting activity of TGFβ but no longer to its initial tumor suppressive activity, reflected by clinical tumor parameters? TGFβ primarily enhances a tumor’s invasive and metastatic potential, which are the major determinants of disease outcome [1–4]. Accordingly, we propose that markers of poor prognosis define an advanced stage in the context of TGFβ signaling. We indeed observed higher odds ratios associated with the Pro-allele in patients with pT2–4 *vs.* pT1 tumors, grade 2 *vs.* grade 1 tumors, and negative *vs.* positive ER status, although these trends were not significant at the 95% confidence level (data not shown). The BCAC study has reported higher odds ratios associated with the Pro-allele in patients with high tumor grade and stage, and negative ER and PR status, although only the latter association was statistically significant [6]. Similarly, the Pro-allele was associated with a reduced risk of early-stage breast cancer, but an increased risk of breast cancer with advanced stages [18]. We observed a significantly younger age at diagnosis of Pro/Pro patients compared to Leu/Leu patients, which could also be due to a faster breast cancer progression associated with the Pro-allele (Figure 1). This novel finding is consistent with a trend towards higher odds ratios associated with the Pro-allele in younger patients in the BCAC study [6].

## 3. Experimental Section

The study population has been described in detail in [27]. Briefly, 276 consecutive female breast cancer patients and 255 controls (patients with benign gynecological lesions and healthy female donors without breast cancer or any other malignancies) were enrolled between 2002 and 2004 at the Department of Obstetrics and Gynecology, Medical University of Vienna (MUV), Austria. Only women of Caucasian background from the same geographical area were included in this study. This study was approved by the institutional review board of the MUV, and written informed consent was obtained from all participants. For technical reasons, the genotype could not be determined for 2 patients and 3 controls. Thus, all further analyses were based on the remaining 526 subjects; their clinical and histopathological characteristics are shown in Table 1.

Genomic DNA was extracted from patients’ blood with the QIAamp DNA Blood Midi kit (Qiagen, Venlo, the Netherlands) per the manufacturer’s instructions. Genotyping of SNP rs1800470 (*TGFβ*1 L10P; Leu10Pro; T + 29C; formerly termed rs1982073) was performed by TaqMan PCR with allele-specific, fluorescently labeled probes following the manufacturer’s instructions (Applied Biosystems, Brunn/Gebirge, Austria; Assay-ID # C_22272997_10). 40 ng of genomic DNA were used per reaction in a total reaction volume of 10 μL.

Serum samples were collected from a subset of the patients and controls of this study between 2003 and 2004 under identical fasting conditions. None of the patients had undergone cancer treatment prior to serum isolation. Following genotyping, all available samples of patients (*n* = 25) and controls (*n* = 26) with the Pro/Pro genotype, and roughly equal numbers of randomly selected samples of the Leu/Leu and Leu/Pro genotypes were subjected to ELISA measurements of TGFβ1 concentrations with the Emax ImmunoAssay System (Promega, Madison, WI, USA). Prior to measurement, diluted serum samples were treated with hydrochloric acid, following the manufacturer’s instructions to release any latent TGFβ1 from complexes with other serum proteins.

Statistical analyses were performed with R version 2.15.1, an open-source language and environment for statistical computing [28]. Potential deviations of the study population from Hardy-Weinberg equilibrium were assessed with Chi-square tests with Yates’ continuity correction. Differences between patients and controls, or between different genotypes, with respect to age or TGFβ1 serum concentrations were analyzed with unpaired, two-sided *t*-tests. Confidence intervals given are 95% mid-P exact confidence intervals, *i.e*., considering all possible configurations of the contingency table that are more extreme than the observed configuration, and half the configurations that are equivalent to the observed one. Likewise, *p*-values shown in Table 2 are mid-P two-tailed exact *p*-values. Associations between the three L10P genotypes and clinical or histopathological characteristics were evaluated with Chi-square tests.

## 4. Conclusions

The current evidence is consistent with a model in which the Pro-allele of *TGFβ*1 is hypermorphic and reduces the overall incidence of breast cancer. However, if a tumor does develop in Pro-allele carriers, it is likely to progress faster to a more advanced stage, with invasive properties and a poor prognosis. Which of these conflicting effects predominates in a particular study likely depends on the specific composition of the study population. Our study had insufficient cases to detect a significant differential risk in subgroup analyses, thus it would be important to perform such analyses in larger studies and/or meta-analyses to better define the clinical subgroups in which the tumor-suppressive *vs.* tumor-promoting activities of TGFβ1 might be present.

## Figures and Tables

**Table 1:** Clinical characteristics of the study population and frequency of the *TGFβ*1 L10P genotypes in the indicated subpopulations.

		Total	Leu/Leu	Leu/Pro	Pro/Pro	*p*-value
All subjects		526	186 (35.4%)	248 (47.1%)	92 (17.5%)	

Patients		274	99 (36.1%)	131 (47.8%)	44 (16.1%)	0.664

Controls		252	87 (34.5%)	117 (46.4%)	48 (19.0%)	

Patient subgroups						

Tumor size	pT1	133	50 (37.6%)	62 (46.6%)	21 (15.8%)	0.586
	pT2	55	20 (36.4%)	27 (49.1%)	8 (14.5%)	
	pT3, pT4	12	3 (25.0%)	5 (41.7%)	4 (33.3%)	
	other, na	74	26 (35.1%)	37 (50.0%)	11 (14.9%)	

Tumor type	ductal	148	53 (35.8%)	71 (48.0%)	24 (16.2%)	0.561
	lobular	48	20 (41.7%)	23 (47.9%)	5 (10.4%)	
	other, na	78	26 (33.3%)	37 (47.4%)	15 (19.2%)	

Stage	I	97	38 (39.2%)	40 (41.2%)	19 (19.6%)	0.290
	II	63	22 (34.9%)	35 (55.6%)	6 (9.5%)	
	III	18	5 (27.8%)	10 (55.6%)	3 (16.7%)	
	other, na	96	34 (35.4%)	46 (47.9%)	16 (16.7%)	

Grade	pG1	42	17 (40.5%)	21 (50.0%)	4 (9.5%)	0.405
	pG2	115	38 (33.0%)	58 (50.4%)	19 (16.5%)	
	pG3	88	36 (40.9%)	35 (39.8%)	17 (19.3%)	
	na	29	8 (27.6%)	17 (58.6%)	4 (13.8%)	

Lymph node status	pN0	143	56 (39.3%)	62 (43.4%)	25 (17.5%)	0.320
	pN+	53	18 (34.0%)	29 (54.7%)	6 (11.3%)	
	na	78	25 (32.1%)	40 (51.3%)	13 (16.7%)	

ER status	pos	201	75 (37.3%)	100 (49.8%)	26 (12.9%)	0.085
	neg	61	21 (34.4%)	25 (41.0%)	15 (24.6%)	
	na	12	3 (25.0%)	6 (50.0%)	3 (25.0%)	

PR status	pos	138	47 (34.1%)	73 (52.9%)	18 (13.0%)	0.130
	neg	117	46 (39.3%)	48 (41.0%)	23 (19.7%)	
	na	19	6 (31.6%)	10 (52.6%)	3 (15.8%)	

HER2 status	pos	51	24 (47.1%)	21 (41.2%)	6 (11.8%)	0.176
	neg	201	67 (33.3%)	99 (49.3%)	35 (17.4%)	
	na	22	8 (36.4%)	11 (50.0%)	3 (13.6%)	

p53 status	pos	57	23 (40.4%)	22 (38.6%)	12 (21.1%)	0.296
	neg	190	68 (35.8%)	94 (49.5%)	28 (14.7%)	
	na	27	8 (29.6%)	15 (55.6%)	4 (14.8%)	

Numbers of patients in each of the indicated subgroups are shown. Numbers in parentheses indicate the fraction of patients in each row with genotypes Leu/Leu, Leu/Pro and Pro/Pro, respectively. na: status not available; ER: estrogen receptor; PR: progesterone receptor. *p*-Values were calculated with chi-square tests of the specified subgroups (excluding na subjects).

**Table 2:** Odds ratios and 95% confidence intervals for *TGFβ*1 L10P genotypes or alleles and breast cancer risk.

Genotypes/Alleles	OR	95% c.i.	*p*-Value
Pro/Pro *vs.* Leu/Leu	0.81	0.49–1.33	0.409
Pro/Pro *vs.* Leu/Pro	0.82	0.51–1.32	0.429
Leu/Pro *vs.* Leu/Leu	0.98	0.67–1.44	0.934
Pro/Pro + Leu/Pro *vs.* Leu/Leu	0.93	0.65–1.33	0.700
Pro/Pro *vs.* Leu/Pro + Leu/Leu	0.81	0.52–1.28	0.368
Pro *vs.* Leu	0.91	0.71–1.16	0.455

Analyses of breast cancer cases *vs.* controls of the indicated genotypes or Pro *vs.* Leu alleles are shown. c.i.: confidence interval.
